# Identification of a novel TLE6 mutation linked to embryonic arrest and limited rescue by mRNA supplementation

**DOI:** 10.3389/fmed.2025.1716251

**Published:** 2025-12-23

**Authors:** Ben Yuan, Tian Xu, Bingbing Luo, Ziqin Lu, Junbiao Mao, Xiumei Wang, Junling Wang

**Affiliations:** 1 Department of Reproductive Medicine, Huangshi Central Hospital, Affiliated Hospital of Hubei Polytechnic University, Huangshi, China; 2 Huangshi Key Laboratory of Assisted Reproduction and Reproductive Medicine, Huangshi, China; 3 Key Laboratory of Animal Embryo Engineering and Molecular Breeding of Hubei Province, Institute of Animal Sciences and Veterinary Medicine, Hubei Academy of Agricultural Sciences, Wuhan, China; 4 Department of Reproductive Medicine, Graduate Joint Training Base (Huangshi Central Hospital), School of Medicine, Wuhan University of Science and Technology, Wuhan, China

**Keywords:** maternal-effect gene, subcortical maternal complex, transducin-like enhancer of split 6, gene variant, embryonic development

## Abstract

**Background:**

The subcortical maternal complex (SCMC) orchestrates early embryogenesis; TLE6, a core SCMC component, stabilizes the complex and regulates cytoskeletal dynamics during the oocyte-to-embryo transition. Pathogenic TLE6 variants are associated with pre-implantation arrest and infertility, but the mutational spectrum and rescue strategies remain incompletely defined. This study aims to describe a case of embryonic arrest associated with a novel homozygous TLE6 variant, elucidate its pathogenic mechanism, and evaluate an mRNA add-back rescue approach.

**Materials and methods:**

A case of primary infertility with repeated IVF failure underwent whole-exome sequencing, followed by confirmatory Sanger sequencing performed in the proband and parents. The variant function was assessed using a minigene splicing assay, Mutalyzer prediction, and structural modeling. A CRISPR/Cas9 Tle6 mouse model was evaluated for developmental consequences. Wild-type Tle6 mRNA was microinjected into mutant zygotes to test its rescue potential.

**Results:**

A novel homozygous splice-region variant in TLE6 (NM_001143986.1: exon7: c.286-7G > A) was identified, with both parents confirmed as heterozygous carriers. The minigene assay showed aberrant splicing with a 5-nucleotide insertion, producing a frameshift and premature truncation. Structural modeling predicted the loss of the C-terminal domain essential for SCMC integrity. The Tle6 mutant mouse recapitulated cleavage-stage arrest with reduced blastocyst formation. Zygotic add-back of wild-type Tle6 mRNA yielded only limited improvement, with persistent developmental failure.

**Conclusion:**

These findings expand the TLE6 variant spectrum and support a loss-of-function mechanism causing embryonic arrest through SCMC disruption. Acute zygotic mRNA supplementation was insufficient to restore developmental competence, indicating the need for alternative or early-stage interventions and informing genetic counseling for affected families.

## Introduction

Mammalian embryogenesis begins with the fusion of the sperm and the oocyte. Current evidence indicates unequal contributions from the two gametes to early development. The sperm that traverses the zona pellucida delivers haploid chromosomes and a subset of mitochondria that are non-essential for embryogenesis (1). In contrast, oocytes accumulate abundant RNAs and proteins; their corresponding genes, that is, maternal-effect genes, provide maternal mRNAs and proteins that are stored in mature oocytes (2). Maternal-effect genes are decisive regulators of pre-implantation development, particularly prior to zygotic genome activation (3). They participate in maternal transcript clearance, epigenetic remodeling, embryonic genome activation, compaction, and establishment of cell polarity, among other events (2). Mutations in these genes can cause early embryonic arrest and are a significant cause of repeated failure in assisted reproductive technology.

In 2008, using mouse models, Li et al. identified the functional subcortical maternal complex (SCMC) in mammals (4). The SCMC is encoded by multiple maternal-effect genes (5–7). Four components, including FLOPED (4), MATER (8), FILIA (9), and TLE6 (10), were initially reported. Additional components have since been validated, including PADI6 (4), NLRP7 (11), NLRP2 (12), and ZBED3 (13). Among known components, FLOPED, MATER, and TLE6 constitute the core of the complex (4, 8, 10), whereas others may be non-core members (14). Loss of core components predominantly arrests embryos at the two-cell stage and leads to complete infertility in female knockout mice (15); loss of non-core components (e.g., FILIA, ZBED3, and NLRP2) delays cleavage-stage development and reduces female fertility (9, 12, 13).

TLE6 (chromosome 19p13.3) contains 17 exons encoding a 572-amino acid protein that scaffolds the SCMC by binding MATER and FILIA via its N-terminal region, thereby maintaining complex stability and regulating early cleavage divisions. It does not affect ovarian development or fertilization per se, although Tle6 deficiency in male mice causes abnormal sperm morphology and reduced motility (16); in female mice, it arrests embryos at the two-cell stage (2). Reported TLE6 mutations (e.g., c.1529C > T/p. S510F) disrupt SCMC assembly and cause embryonic lethality (17), clinically manifesting as recurrent *in vitro* fertilization (IVF) failure or early miscarriage (18). Many variants cluster in functional domains (e.g., WD40 repeats) and impair protein–protein interactions. Genetically, approximately 10–15% of unexplained recurrent IVF failure is linked to maternal-effect gene defects (19). Current clinical screening focuses on known loci; 11 TLE6 variant types have been implicated in infertility, but they explain only a fraction of cases (16, 20, 21). Identifying novel variants is therefore essential for precise diagnosis.

In this study, we report a patient who underwent two *in vitro* fertilization (IVF) cycles with no usable day-3 embryos. Whole-exome sequencing identified a homozygous splice-region variant in TLE6 (NM_001143986.1: exon7: c.286-7G > A) that was absent from population and clinical variant databases (e.g., gnomAD and ClinVar). We expand the TLE6 variant spectrum and interrogate pathogenicity through a tiered approach combining minigene splicing analysis and structural inference with a CRISPR/Cas9 mouse model. Finally, we evaluate an RNA add-back strategy in mutant zygotes to probe the feasibility, constraints, and timing of intervention for maternal-effect gene defects.

## Materials and methods

### Clinical case

A woman presented with primary infertility, having failed to conceive after 1 year of unprotected intercourse; subsequently, she underwent two IVF attempts. Fertilization was normal, but embryos exhibited heavy fragmentation and developmental arrest after day 2. No usable day-3 embryos were obtained in either cycle. The partner’s semen analysis was normal. Both partners had normal somatic and reproductive development. The study was approved by the Ethics Committee of Huangshi Central Hospital. Written informed consent was obtained from the patient.

### IVF stimulation protocols

First cycle: A gonadotropin-releasing hormone (GnRH) antagonist protocol was applied. Controlled ovarian stimulation was initiated with human menopausal gonadotropin (HMG, 150 IU; Lizhu Pharmaceutical, China) combined with urofollitropin (150 IU; Lizhu Pharmaceutical, China) for 10 consecutive days. From stimulation day 6, ganirelix acetate (0.25 mg; Vetter Pharma-Fertigung GmbH & Co. KG, Germany) was administered daily for 6 days. Final oocyte maturation was triggered with 10,000 IU of human chorionic gonadotropin (hCG; Lizhu Pharmaceutical, China), and oocyte retrieval was performed 36-h post-trigger.

Second cycle: A progestin-primed ovarian stimulation (PPOS) protocol was applied. Controlled ovarian stimulation was performed using recombinant follicle-stimulating hormone-β (rFSH-β, 225 IU; Vetter Pharma-Fertigung GmbH & Co. KG, Germany) in combination with human menopausal gonadotropin (HMG, 75 IU; Lizhu Pharmaceutical, China) for 9 consecutive days. Oral medroxyprogesterone acetate (10 mg daily) was administered during the stimulation. Final oocyte maturation was triggered with triptorelin acetate (0.2 mg; Ferring Pharmaceuticals, Switzerland) plus hCG (2000 IU; Lizhu Pharmaceutical, China). Oocyte retrieval was performed 36-h post-trigger.

### Genetic testing

Peripheral blood DNA from the proband and her parents underwent whole-exome sequencing (WES) using the SureSelect XT V6 Human All Exon kit (Agilent, Santa Clara, CA, USA) and was sequenced on an Illumina NextSeq 500 (Illumina, San Diego, CA, USA). Reads were aligned to hg19 (UCSC) using the Burrows–Wheeler Aligner (BWA); Single-nucleotide variants (SNVs) and insertions/deletions (InDels) were identified with Genome Analysis Toolkit (GATK) HaplotypeCaller; variants were annotated using ClinVar, the Human Gene Mutation Database (HGMD), Sorting Intolerant From Tolerant (SIFT), Polymorphism Phenotyping (PolyPhen), local databases, and in-house pipelines. Copy-number analysis used Exome Hidden Markov Model (XHMM) and Copy Number Detection by Local Assembly and Mapping of Sequence Signals (CLAMMS). Sanger sequencing confirmed variant and parental heterozygosity. A mutalyzer was used to predict frameshift effects.

### Pathogenicity assessment

Genomic fragments encompassing exon 6, intron 6, and part of exon 7 (wild-type or mutant) were cloned into pCDH-MCS-T2A-puroR and transfected into 293 T cells. At 36 h, RNA was extracted, reverse-transcribed, and PCR-amplified for Sanger sequencing. Mixed peaks in mutant cDNA indicated splicing defects. To enrich successfully transfected cells, puromycin (2 μg/ml) was applied at 48 h after transfection, and surviving cells were monitored under the microscope for morphology and viability. Differences between wild-type and mutant constructs were assessed by cell viability counts and microscopic evaluation, providing functional evidence for the deleterious impact of the splice-site mutation.

### 3D modeling of TLE6

The three-dimensional structure of human TLE6 was predicted using AlphaFold to evaluate the consequences of the splice-site mutation. The UniProt reference sequence was submitted to the AlphaFold pipeline, and the top-ranked model was selected based on: predicted local distance difference test (pLDDT) scores and predicted aligned error (PAE) profiles. To model the mutant, an alternative sequence reflecting the aberrant splicing event (c.286-7G > A) was generated, introducing a premature termination codon near Val96 and resulting in the loss of the C-terminal domain. The truncated sequence was analyzed under the same AlphaFold workflow. Wild-type and mutant models were visualized and superimposed using PyMOL and ChimeraX to assess global secondary and tertiary structures as well as local conformational deviations. Special attention was given to the Leu88-Val96 region, where structural displacement and abnormal helix formation were quantified, and to the C-terminal region, which is implicated in protein–protein interactions within the subcortical maternal complex (SCMC). This combined modeling and visualization approach enabled structural inference of domain loss, altered folding, and the resulting destabilization of TLE6.

### Animals and ethical approval

All animal experiments were performed at the Laboratory Animal Center of Huazhong Agricultural University (Wuhan, China). C57BL/6 J mice were obtained from the Model Animal Research Platform of Huazhong Agricultural University. Experimental protocols were reviewed and approved by the Animal Ethics Committee of Huazhong Agricultural University, and all procedures were conducted in accordance with institutional guidelines for animal care and use.

### Superovulation and mating

Healthy C57BL/6 J female mice (8–12 weeks old) were subjected to superovulation by intraperitoneal injection of pregnant mare serum gonadotropin (PMSG, 5 IU), followed 48 h later by human chorionic gonadotropin (hCG, 5 IU). Female mice were then housed overnight with C57BL/6 J male mice (≥8 weeks old). The presence of vaginal plugs was checked the following morning, and plug-positive female mice were selected for embryo collection.

### Collection and preparation of zygotes

Approximately 20 h after hCG injection, fertilized eggs were harvested from the oviductal ampullae of plugged female mice. The surrounding cumulus cells were removed by treatment with 0.1% hyaluronidase in M2 medium, and zygotes were thoroughly washed before use. Clean zygotes were transferred into G1-PLUS medium (Vitrolife) under mineral oil and cultured in a 37 °C incubator with 5% CO₂ until microinjection.

### CRISPR/Cas9 microinjection

To generate Tle6 knockout mice, four single-guide RNAs (sgRNAs) targeting the exon regions of Tle6 were designed using CRISPR/Cas9 technology. The sgRNA sequences were as follows: mouse_Tle6_KO_sg1, 5′-TACTGAAGCGCTCTATCGCC-3′; mouse_Tle6_KO_sg2, 5′-TCATCTTGTTCCTCTCCAGG-3′. The sgRNAs and Cas9 nuclease were synthesized by *in vitro* transcription and purified before use. A CRISPR/Cas9 mixture (final concentration: Cas9 protein 50 ng/μl; sgRNA 20 ng/μl) was then microinjected into the cytoplasm of one-cell-stage zygotes using a micromanipulator under an inverted microscope. After recovery, approximately 15–20 injected zygotes were surgically transferred into the oviducts of pseudopregnant recipient female mice at 0.5 days post coitum.

### Breeding and genotyping

Newborn F0 pups were tail-clipped for genomic DNA extraction. Genotyping was performed by PCR amplification using the primer pair 5′-TCAGCAAGGCTCCATCCTAACT-3′ (forward) and 5′-CAGCCCCAACACCAATACTCAT-3′ (reverse). Each PCR reaction was performed in a 25-μl volume containing 50–100 ng of genomic DNA, 0.4 μM of each primer, and 12.5 μl of 2 × Taq PCR Master Mix (Tiangen, China). The thermal cycling conditions were as follows: initial denaturation at 95 °C for 5 min; 35 cycles of denaturation at 95 °C for 30 s, annealing at 60 °C for 30 s, and extension at 72 °C for 40 s, followed by a final extension at 72 °C for 5 min. PCR products were analyzed by agarose gel electrophoresis and confirmed by Sanger sequencing to verify the targeted indels. F0 founder animals carrying Tle6 mutations were crossed with wild-type C57BL/6 J mice to generate F1 heterozygotes (^+/−^). F1 mice derived from the same F0 founder and carrying the same genotype were intercrossed to obtain F2 offspring. Genotyping of F2 mice identified Tle6 homozygous knockouts (^−/−^), heterozygotes (^+/−^), and wild-type (^+/+^) littermates. The positions of the genotyping primers and sgRNA target sites for Tle6 are shown in Supplementary Figure S1.

### RNA rescue experiment

A Tle6 overexpression construct was prepared for *in vitro* transcription to produce wild-type Tle6 mRNA. The coding sequence of Tle6 was amplified by PCR, and a T7 promoter sequence was introduced immediately upstream of the ATG start codon to enable transcription. *In vitro* transcription was performed using the MEGAscript™ T7 Transcription Kit (Cat. No. AM1354, Thermo Fisher Scientific Waltham, USA), according to manufacturer’s instructions. The transcribed mRNA was subsequently purified by LiCl precipitation to remove unincorporated nucleotides and enzymes, and the RNA integrity was verified by agarose gel electrophoresis. From TLE6^+/+^, ^+/−^, and ^−/−^ females (n = 2 per genotype), 30–60 oocytes/genotype were collected and fertilized *in vitro* using wild-type spermatozoa from healthy C57BL/6 J male mice. Four hours post-fertilization, wild-type Tle6 mRNA (500 ng/μl) was injected into one half of the zygotes; the remainder served as uninjected controls. Then, developmental progression and safety were recorded.

### Statistical analysis

The data analysis was performed using GraphPad Prism Version 9.0 (GraphPad Software). All results were expressed as mean ± SEM. Significant differences between groups were analyzed using a two-way ANOVA followed by Bonferroni’s multiple comparison tests. *A *p*-value of <0.05 was considered statistically significant.

## Results

### Clinical characteristics

The proband was a 37-year-old woman with primary infertility. She reported regular menstrual cycles and normal ovulation. Hysterosalpingography indicated patent but poorly conductive fallopian tubes. Ovarian reserve assessment revealed normal parameters, and her partner’s semen analyses were unremarkable. No family history of infertility or genetic disorders was reported.

### IVF cycle outcomes

**Figure 1:**
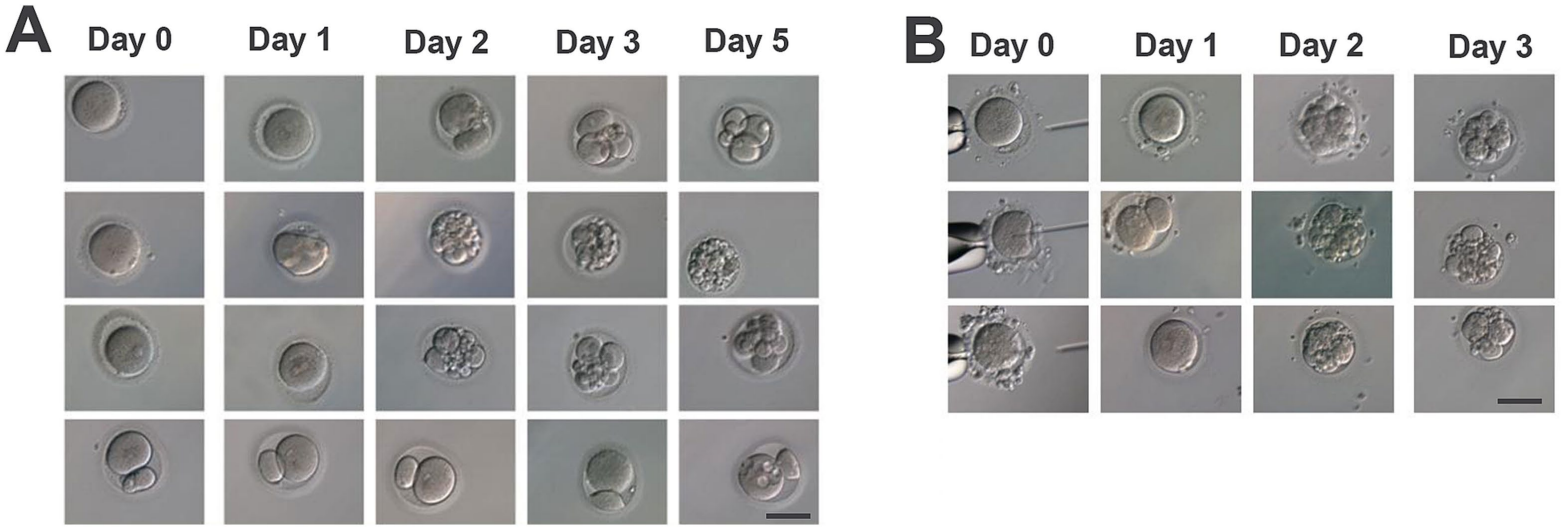
Embryo development in two assisted reproduction cycles of the patient. **(A)** Cycle 1: Oocyte retrieval was performed 36 h after hCG, yielding 6 oocytes (all MII; one with a giant polar body). After IVF, 4 zygotes showed normal fertilization. By Day 3, embryos exhibited developmental arrest at the 2–3-cell stage; one embryo reached the 5-cell stage with pronounced fragmentation. Extended culture toward the blastocyst stage resulted in degeneration. **(B)** Cycle 2: Oocyte retrieval was performed 36 h after hCG, yielding 8 oocytes (3 immature/degenerate and 5 MII). Following ICSI, 2 zygotes showed normal fertilization. By Day 3, embryos arrested at the 4-cell stage with substantial fragmentation. Images were captured under a light microscope; panel labels correspond to the indicated stages. Each row depicts the developmental progression of embryos derived from individual oocytes. Scale bar = 100 μm.

For the first cycle, six oocytes were retrieved, all at the metaphase II (MII) stage, including one with a giant polar body. Four oocytes fertilized normally; however, all embryos exhibited developmental arrest at the 2–3-cell stage with severe fragmentation. One embryo briefly reached the five-cell stage but degenerated during blastocyst culture (Figure 1; Table 1).

**Table 1:** Clinical characteristics and outcomes across two ART cycles in the patient carrying a TLE6 variant.

Age (years)	Duration of infertility (years)	Cycle	Fertilization method	Oocytes retrieved	MII oocytes	Normally fertilized (2PN)	Usable day-3 embryos	Semen analysis	Donor-oocyte cycles	Donor-oocyte clinical outcome
37	2	1	IVF	6	6	4	0	Normal	1	Live birth
		2	ICSI	8	5	2	0	Normal		

ART, assisted reproductive technology; MII, metaphase II; 2PN, two-pronuclear; IVF, in vitro fertilization; ICSI, intracytoplasmic sperm injection. Dashes indicate not applicable.

For the second cycle, eight oocytes were retrieved, three of which were immature or degenerated, leaving five MII oocytes. After Intracytoplasmic Sperm Injection (ICSI), the two fertilized normally, but both embryos were arrested at the four-cell stage with marked fragmentation. No transferable embryos were obtained (Figure 1; Table 1).

### Genetic analysis

Since both IVF cycles resulted in embryonic developmental arrest with marked fragmentation and no usable day-3 embryos, WES was performed. An analysis revealed a homozygous splice-region variant in the TLE6 gene on chromosome 19 (NM_001143986.1: exon7: c.286-7G > A). Pathogenic variants in TLE6 are known to cause autosomal recessive pre-implantation embryonic lethality type 1 (MIM #616814). In affected female mice, ovulation occurs normally and oocytes appear morphologically intact, but fertilization and subsequent embryonic cleavage are severely compromised. The identified variant has not been previously reported in the literature. Previous studies have demonstrated that TLE6-associated embryonic lethality is mediated by loss-of-function mechanisms.

**Figure 2:**
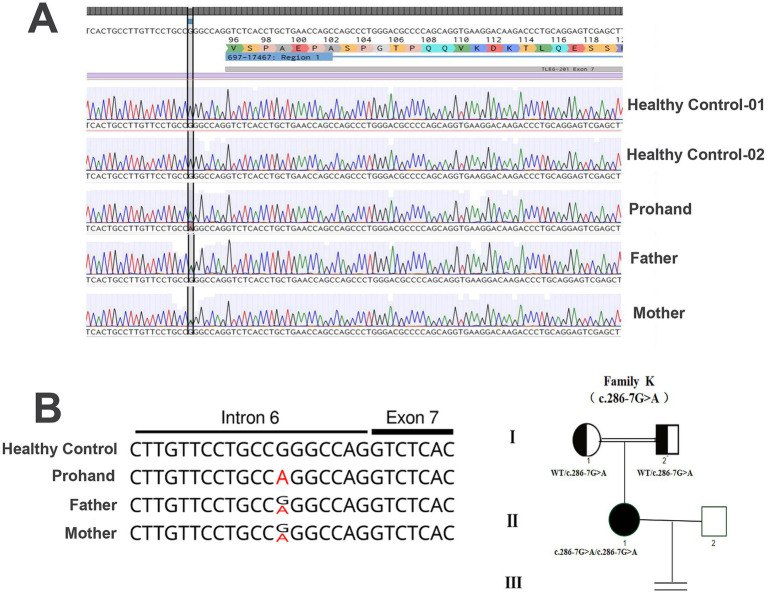
TLE6 genotypes of the patient and her parents. **(A)** The patient carries a homozygous G → A substitution at position −7 upstream of exon 7 in TLE6; both parents are heterozygous. **(B)** Pedigree and TLE6 variant analysis. Squares indicate male mice; circles indicate female mice; filled symbols denote affected individuals; open symbols denote unaffected individuals; the equals sign indicates no offspring. Sanger sequencing of TLE6 coding exons in family members reveals the homozygous variant in the proband and heterozygous variants in both parents.

According to the American College of Medical Genetics and Genomics (ACMG) guidelines, this variant was classified as a variant of uncertain significance. To validate the finding, Sanger sequencing of peripheral blood samples from the proband and her parents confirmed that both parents carried the same heterozygous mutation at the TLE6 locus, which was consistent with an autosomal recessive inheritance pattern (Table 2). A detailed family history revealed consanguinity, and the pedigree analysis further supported the genetic segregation pattern (Figure 2). Mutalyzer prediction indicated that the variant introduces a frameshift mutation resulting from aberrant splicing.

**Table 2:** Whole-exome sequencing (WES) report for the patient.

Gene	Chromosome	Position	dbSNP ID	Variant nomenclature	gnomAD EAS frequency	ACMG classification	Zygosity (proband)	Mother	Father
TLE6	Chr19	2,986,974	rs756140063	TLE6: NM_001143986.1: exon7: c.286-7G > A	0	Variant of uncertain clinical significance	Homozygous	Heterozygous	Heterozygous

### Structural modeling

**Figure 3:**
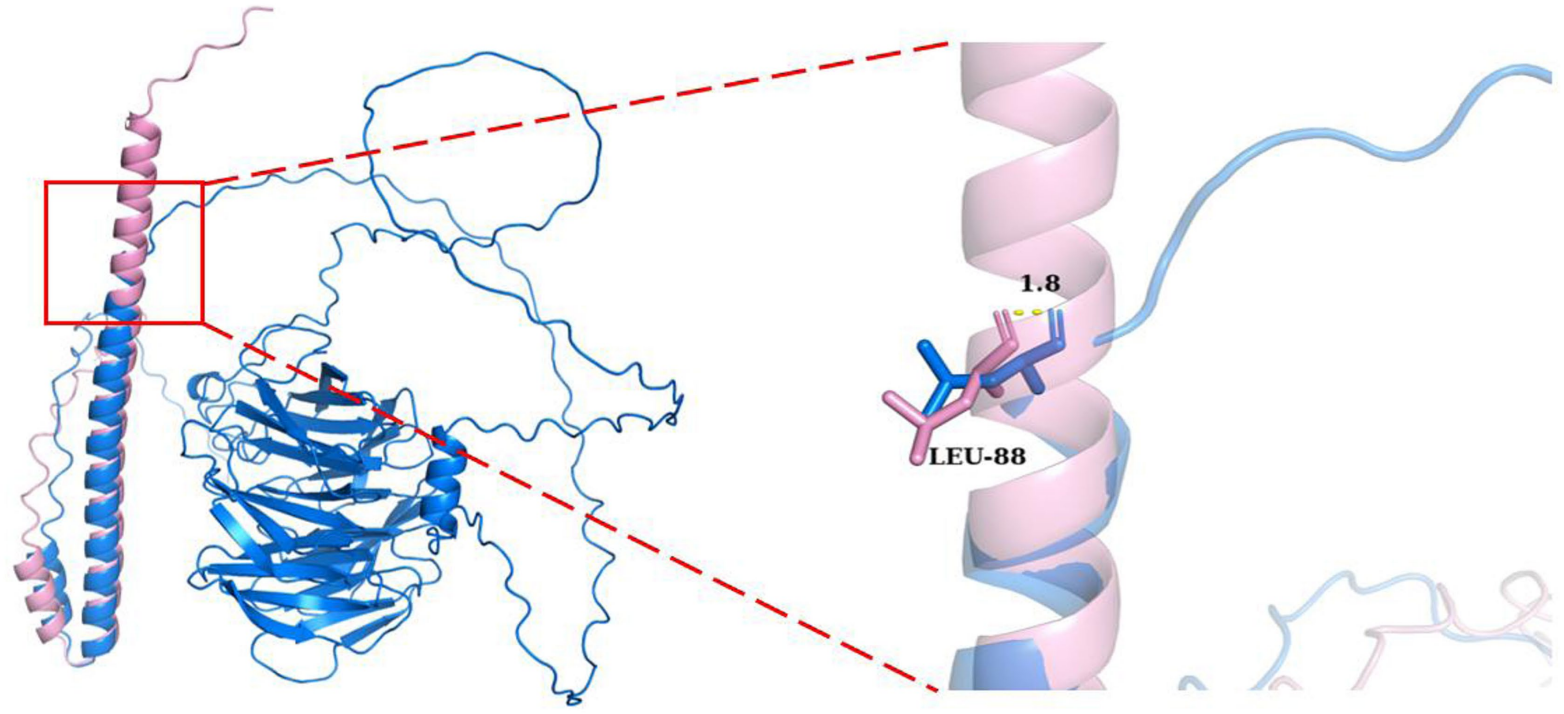
Structural comparison of TLE6 wild type (blue) and mutant (pink). The mutant exhibits loss of the C-terminal domain due to aberrant splicing, retaining only part of the N-terminal structure. Right: close-up of the Leu88 region. The mutant forms a short α-helix at this site, whereas the wild type is irregular/coil. The backbone conformations diverge by ~1.8 Å at Leu88, highlighting substantial local perturbation and altered folding.

To assess the functional consequences, the three-dimensional structure of the TLE6 protein was modeled (Figure 3). The wild-type protein displayed a stable β-barrel core surrounded by multiple α-helices, providing a robust structural framework essential for its biological function. In contrast, the mutant protein retained only a short N-terminal α-helix, while the C-terminal region collapsed, lacking defined secondary structural elements and stable tertiary conformation. This conformational disruption was attributed to aberrant splicing that introduced a premature termination codon near residue Val96, yielding a truncated polypeptide chain devoid of the complete C-terminal domain. The C-terminal domain of TLE6 is known to mediate critical protein–protein interactions involved in cytoskeletal reorganization and signal transduction, both essential for the oocyte-to-embryo transition. Loss of this domain destabilizes the overall protein, leading to rapid degradation and functional insufficiency.

At the local structural level, the mutant protein exhibited formation of an abnormal α-helix near Leu88, whereas the wild-type protein displayed a flexible coil structure at this position. Although the mutation originated at Val96, conformational deviation began as early as Leu88, with an estimated 1.8 Å backbone displacement that progressively worsened downstream. This aberrant local folding disrupted the native conformational pathway, likely impairing global protein stability and molecular function.

### Minigene assay

**Figure 4:**
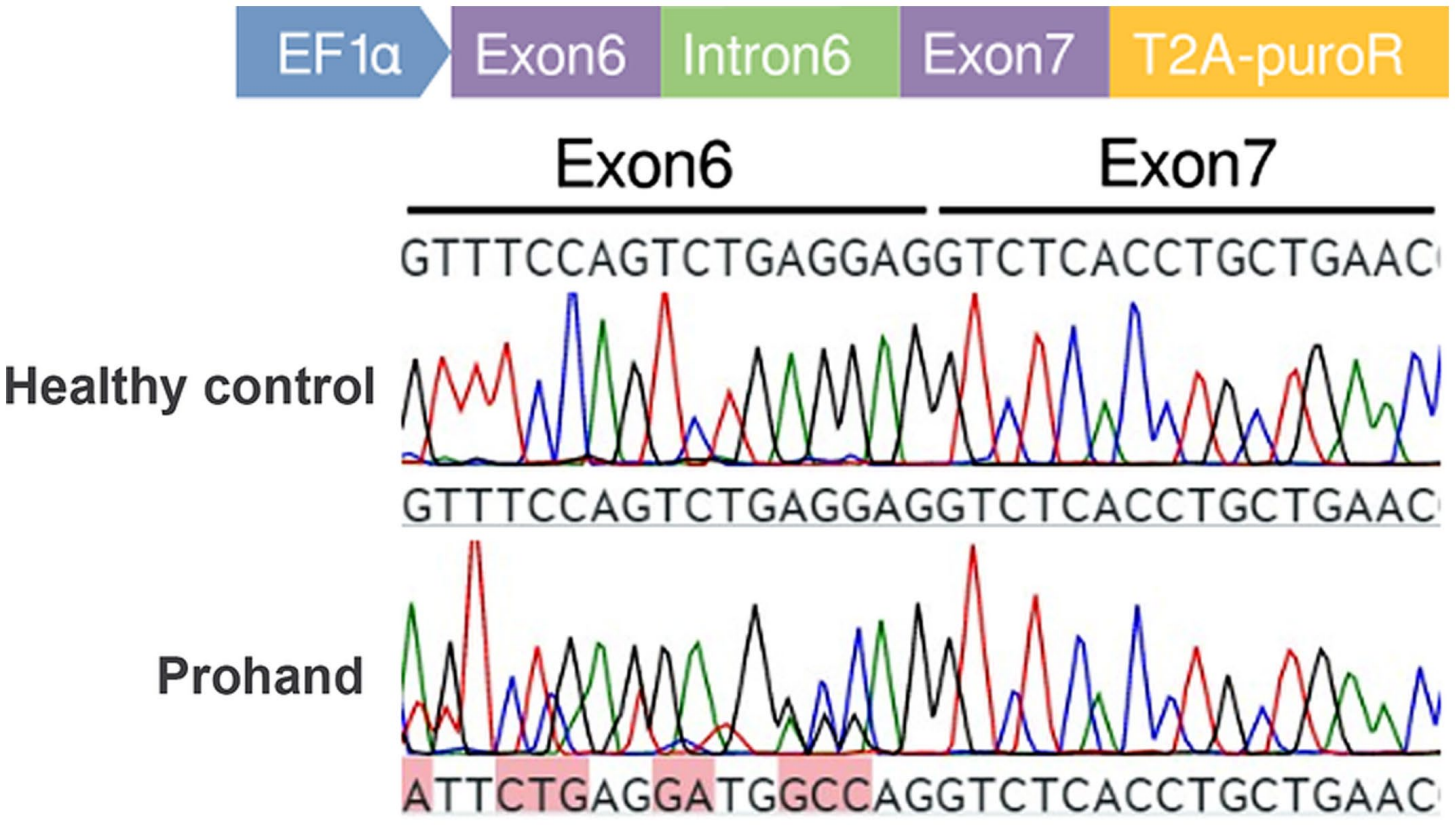
Minigene assay schematic and transcript cDNA analysis. Construction of control and mutant TLE6 minigene expression vectors and transfection into 293 T cells; RNA was harvested at 48 h and reverse-transcribed to cDNA. PCR amplification and Sanger sequencing of the target fragment show peak overlap (“mixed peaks”) in the mutant group.

To investigate the effect of the identified splice-site variant on TLE6 mRNA splicing, a minigene assay was performed. Genomic DNA fragments encompassing exons 6 and 7 of TLE6 were cloned into the pCDH-MCS-T2A-puroR vector, which was subsequently transfected into 293 T cells. After 36 h, total RNA was extracted, reverse-transcribed into cDNA, and analyzed by PCR followed by Sanger sequencing to evaluate splicing patterns in the mutant and control constructs (Figure 4).

**Figure 5:**
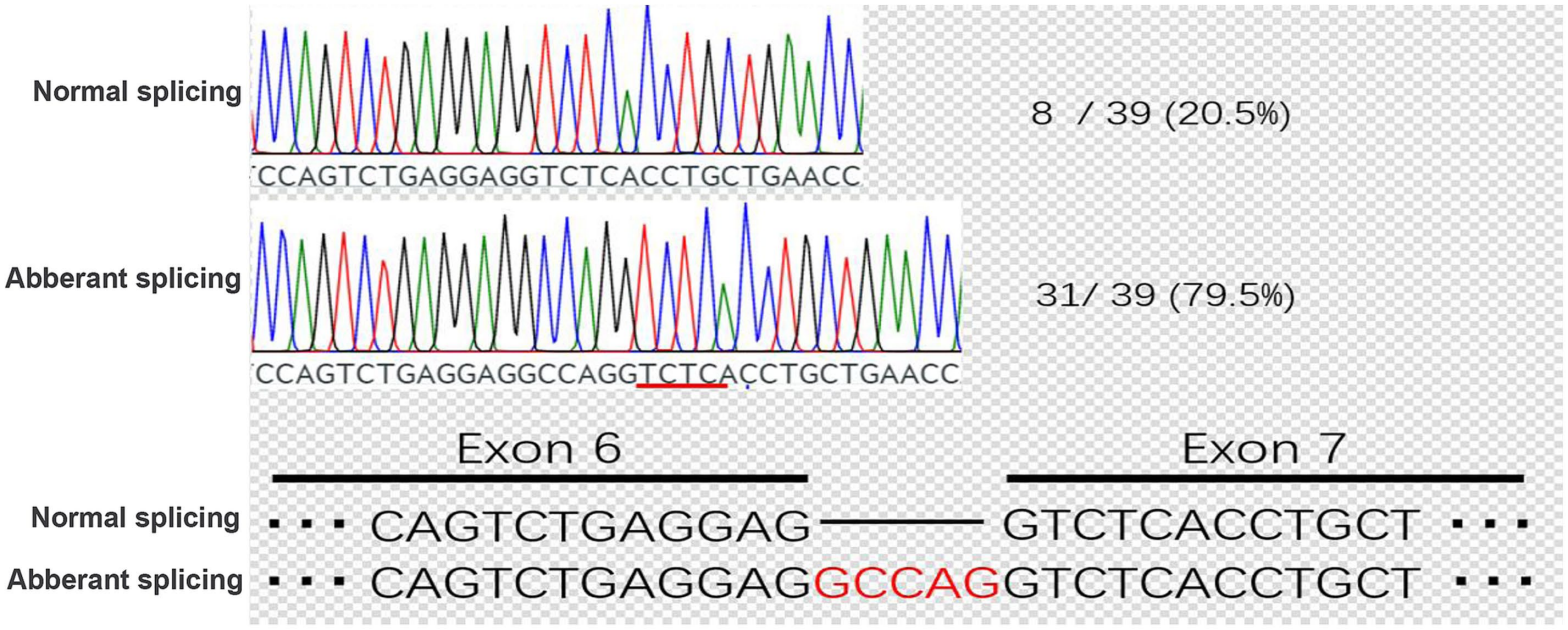
Cloning and sequencing of patient TLE6 transcript cDNA. PCR products from the KX group were ligated into the pMD19-T vector, single colonies were sequenced, and genotype proportions were tallied. In the KX group, correctly spliced mRNA accounted for 20.5%, whereas aberrant splicing caused by the point mutation accounted for 79.5%.

Sequencing of PCR products revealed that the mutant construct generated two distinct transcripts. One transcript was identical to the wild-type control, accounting for 20.5% of clones. The second transcript, representing the majority (79.5%), exhibited aberrant splicing characterized by a 5-nucleotide insertion, resulting in a frameshift mutation and premature truncation (Figure 5). The presence of the correctly spliced transcript in a minority of clones may reflect low-level endogenous TLE6 expression in 293 T cells, suggesting that a fraction of the apparent wild-type product could represent background amplification rather than accurate splicing of the mutant construct.

### Mouse genotype verification

A Tle6 knockout mouse was generated using CRISPR/Cas9 genome editing. Genotypes of F2 offspring were determined by PCR amplification of the targeted locus followed by agarose gel electrophoresis. As expected, wild-type animals yielded a single band corresponding to the wild-type allele, heterozygous animals displayed two bands representing both the wild-type and mutant alleles, and homozygous mutants exhibited a single mutant-specific band. Representative results are shown in Supplementary Figure S2, in which sample 4 corresponds to a Tle6^−/−^ homozygote, confirming successful establishment of the knockout line. Detailed genotypic distribution data of the F2 offspring were not recorded in this study; therefore, the Mendelian ratio among Tle6^+/+^, Tle6^+/−^, and Tle6^−/−^ genotypes could not be assessed.

### RNA rescue outcome

**Figure 6:**
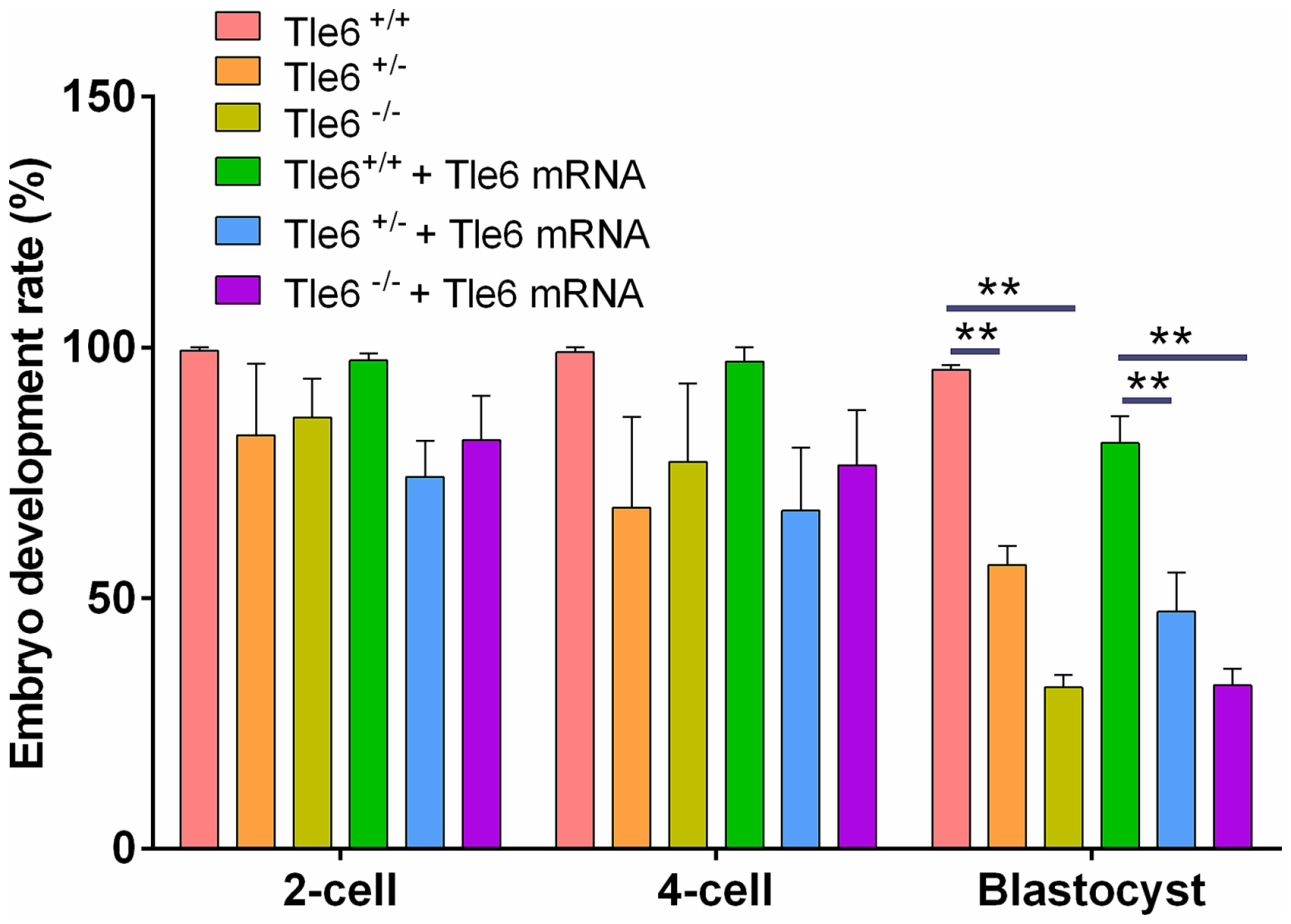
Developmental rates of Tle6-knockout embryos with microinjection of wild-type Tle6 mRNA. Developmental progression of embryos derived from Tle6 ^+/+^, Tle6 ^+/−^, and Tle6^−/−^ maternal genotypes (^m+/+^, ^m+/−^, and ^m−/−^) fertilized *in vitro* with wild-type spermatozoa. Embryos were cultured either without or with microinjection of wild-type Tle6 mRNA at the zygote stage. Bars show the percentage of embryos reaching the 2-cell, 4-cell, and blastocyst stages at the indicated time points. **p < 0.01 and ***p < 0.001.

Using the CRISPR/Cas9 Tle6-knockout mouse line, we observed normal postnatal growth; however, female mutants exhibited abnormal post-fertilization embryonic development, characterized by a reduction in blastocyst formation and an increased proportion of cleavage-stage arrest, indicating that Tle6 is essential for early embryogenesis (Figure 6). To evaluate a potential rescue strategy, wild-type Tle6 mRNA was microinjected into zygotes derived from Tle6-mutant female mice, and embryonic development and offspring outcomes were monitored. mRNA supplementation produced only limited improvement, with no meaningful recovery of developmental competence after fertilization; the blastocyst yield remained low, and overall development failure persisted. These findings suggest that acute zygotic add-back of Tle6 mRNA is insufficient to overcome the developmental deficits caused by maternal Tle6 loss (Figure 6).

## Discussion

This study identifies a previously unreported homozygous splice-region variant in TLE6 on human chromosome 19 (NM_001143986.1: exon7: c.286-7G > A) and links it to early embryonic developmental arrest. The proband’s parents were consanguineous, and both carried the same heterozygous variant, supporting an autosomal recessive mode of inheritance. A minigene assay demonstrated that the variant induces aberrant splicing, generating a frameshift and a premature termination codon, which is expected to lead to nonsense-mediated mRNA decay and/or production of a truncated, non-functional protein, thereby impairing TLE6 synthesis and function and culminating in embryonic arrest (22). These data establish a direct association between TLE6 loss of function and human embryonic developmental failure and expand our current understanding of the molecular control of early human development. Notably, whereas Tle6 knockout in mice typically arrests development at the two-cell stage, several embryos in our patient progressed to the 4–5-cell stage, suggesting species-specific timing windows or compensatory mechanisms in humans (23). Recent evidence suggests that SCMC is constructed during oocyte growth prior to the germinal vesicle (GV) stage rather than after fertilization. Jentoft et al. demonstrated that mammalian oocytes store essential maternal proteins in cytoplasmic lattices before GV breakdown, providing the molecular foundation for subsequent SCMC assembly (24). This finding indicates that appropriate timing is critical for SCMC formation.

TLE6 is a member of the Groucho/TLE co-repressor family with a maternal-effect expression pattern (2). Unlike other TLE paralogs, TLE6 is characterized by a prominent C-terminal WD40 repeat domain and lacks the N-terminal Q domain, a structural distinction with functional implications (25). TLE6 is highly expressed in oocytes and early embryos (26), where it participates in early developmental control, including cross-talk with Notch, Wnt, and transforming growth factor-beta (TGF) pathways implicated in organogenesis and tumorigenesis. Crucially, TLE6 interacts with other SCMC components to stabilize the complex and regulate early cleavage divisions; disruption typically yields arrest around the two-cell stage (10, 27). Mechanistically, core SCMC constituents, including TLE6, stabilize 14–3-3 proteins and help fine-tune CDC25B activity, ensuring appropriate MPF activation and mitotic entry during the early embryonic cycles; perturbation of these interactions can derail the embryonic cell cycle (28). In this framework, the c.286-7G > A variant likely destabilizes the SCMC and/or its signaling outputs, thereby blocking pre-implantation development.

Direct mRNA add-back to rescue embryonic development in TLE6-deficient embryos has not been reported previously, although partial rescue by maternal mRNA or recombinant protein has been shown for other SCMC components (e.g., NLRP5/MATER and FILIA) and maternal-effect genes. For example, maternal BCAS2 deficiency compromises embryonic DNA repair; wild-type BCAS2 RNA supplementation improves genome stability (29). Similarly, for non-SCMC targets, TPX2 mRNA delivery can restore spindle architecture and chromosomal euploidy in models with spindle assembly defects (30); and our study on ELL3 shows that reconstructing the KIF11 complex can partially restore spindle function, underscoring the principle that targeted restoration of maternal factors can be beneficial (31). In our study, wild-type Tle6 mRNA microinjection into zygotes from Tle6-mutant mice produced only limited improvement in post-fertilization development. Potential explanations include mRNA stability, delivery efficiency, dose, and timing relative to the narrow window of maternal-to-zygotic transition, and the possibility that restoring a single SCMC component is insufficient when overall complex integrity or downstream pathways are compromised. Future optimization could involve modified mRNA chemistry, improved delivery kinetics, earlier intervention (oocyte stage), or combinatorial restoration of multiple SCMC members. Consanguinity substantially increases the risk of recessive disorders; thus, genetic counseling is warranted for at-risk couples. In this case, donor-oocyte IVF resulted in a healthy singleton live birth, indicating that oocyte donation is a viable reproductive option for patients with recurrent ART failure attributed to TLE6 variants.

There are limitations to our study. Mechanistic validation was performed on mouse models, which may not fully recapitulate human regulatory dynamics. The precise molecular interfaces through which the human TLE6 variant disrupts SCMC stability and signaling remain to be mapped. The embryo rescue experiments yielded modest gains; additional studies are needed to refine cargo design, dosing, and timing and to test multi-target strategies that address the networked nature of early embryogenesis. Another limitation of this study is the absence of a buffer-injected control group to evaluate the potential influence of the microinjection procedure itself on preimplantation development, and we acknowledge that including such a group would provide additional experimental rigor. Future studies incorporating both uninjected and buffer-injected controls will help to further validate the specificity of the observed effects of TLE6 mRNA injection. Interestingly, approximately 30% of embryos from Tle6^−^/^−^ female mice developed to the blastocyst stage, differing from the complete two-cell arrest reported previously (10). This discrepancy could be attributed to differences in deletion sites, residual Tle6 expression, or genetic background. Partial compensation by other subcortical maternal complex components may also contribute. This variation highlights a limitation of the present study, as precise deletion boundaries and potential compensatory mechanisms were not fully characterized.

## Conclusion

Our integrative analyses—spanning clinical observation, whole-exome sequencing, splicing validation, structural modeling, and a murine knockout model—demonstrate that a novel homozygous TLE6 splice-region variant (NM_001143986.1: exon7: c.286-7G > A) causes embryonic arrest, most plausibly by destabilizing SCMC. Although acute zygotic add-back of wild-type Tle6 mRNA yielded only limited developmental improvement, these findings expand the mutational spectrum of TLE6, underscore the centrality of the SCMC in human pre-implantation development, and provide a molecular diagnosis that is directly relevant to genetic counseling and reproductive decision-making in families with maternal-effect gene defects. Future studies should delineate the TLE6 interactome, define the temporal dosing window for therapeutic augmentation, and evaluate enhanced or combinatorial rescue approaches (e.g., optimized mRNA chemistry, earlier oocyte-stage delivery, or multi-component SCMC restoration). Such mechanism-guided strategies may ultimately improve developmental competence in affected embryos and inform precision interventions at the early stages of human development.

## Data Availability

he original contributions presented in the study are publicly available. This data can be found here: https://www.ncbi.nlm.nih.gov/sra/PRJNA1376516, Accession No.: PRJNA1376516.
