# Supplementary material for: Identification of a novel TLE6 mutation linked to embryonic arrest and limited rescue by mRNA supplementation
Source: Front Med (Lausanne). 2025 Dec 23;12:1716251. doi: 10.3389/fmed.2025.1716251 (PMC12771770; doi:10.3389/fmed.2025.1716251)
Supplement: Supplementary file 1 [file Data_Sheet_1.docx]

Supplementary Material

## Supplementary Figures

**
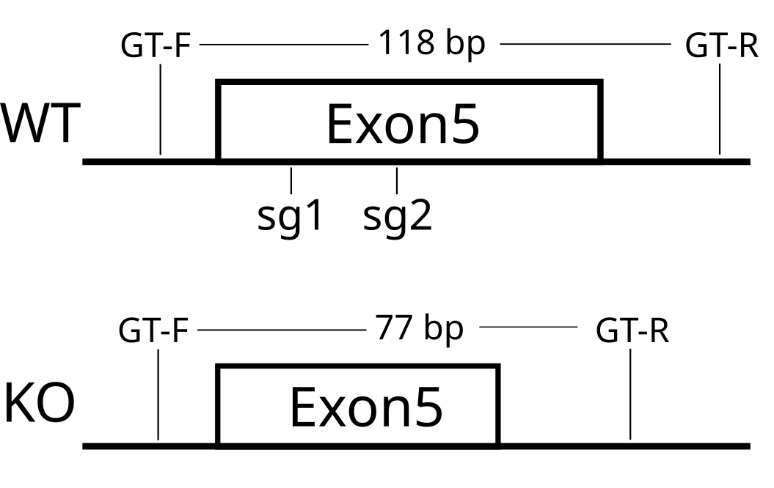
**

**Supplementary Figure 1.** Schematic draw showing the positions of the genotyping primers and sgRNA target site for Tle6.


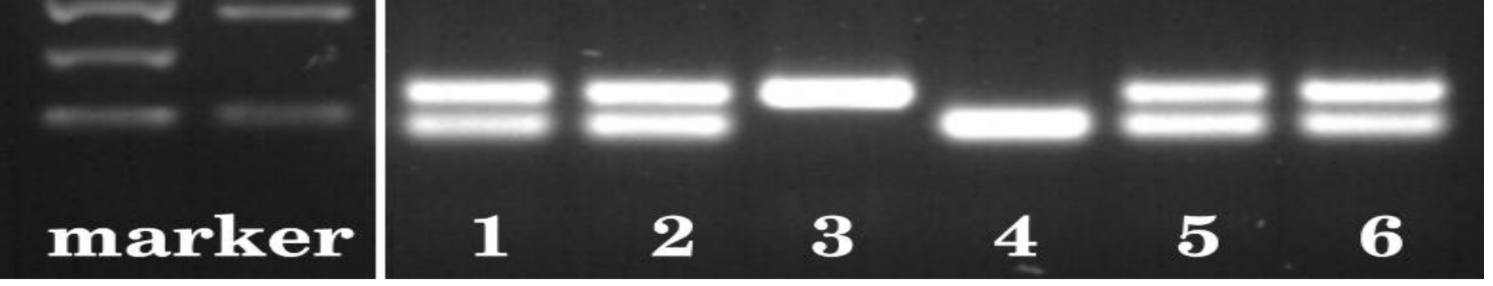


**Supplementary Figure 2.** Genotyping of Tle6 knockout mice. PCR-based genotyping results showing wild-type (WT, ^+/+^), heterozygous (HT, ^+/−^), and homozygous mutant (HM, ^−/−^) alleles. WT samples display a single band corresponding to the wild-type allele, HT samples display two bands (wild-type and mutant alleles), and HM samples display only the mutant band. Lanes 1, 2, 5, and 6 represent heterozygotes (^+/−^); lane 3 represents a wild-type control (^+/+^); and lane 4 corresponds to a homozygous knockout mouse (^−/−^).
